# Fabrication of a Microneedle/CNT Hierarchical Micro/Nano Surface Electrochemical Sensor and Its *In-Vitro* Glucose Sensing Characterization

**DOI:** 10.3390/s131216672

**Published:** 2013-12-04

**Authors:** Youngsam Yoon, Gil S. Lee, Koangki Yoo, Jeong-Bong Lee

**Affiliations:** 1 Department of Electrical Engineering, the University of Texas at Dallas, 800 W. Campbell Rd., Richardson, TX 75080, USA; E-Mails: yyoon4@gmail.com (Y.Y.); gslee@utdallas.edu (G.S.L.); 2 Department of Information and Communication Engineering, Hanbat National University, 125 Dongseodaero, Yuseong-Gu, Daejeon 305-719, Korea; E-Mail: kkryoo@gmail.com

**Keywords:** microneedle, glucose sensing, nanoparticle

## Abstract

We report fabrication of a microneedle-based three-electrode integrated electrochemical sensor and *in-vitro* characterization of this sensor for glucose sensing applications. A piece of silicon was sequentially dry and wet etched to form a 15 × 15 array of tall (approximately 380 μm) sharp silicon microneedles. Iron catalyst was deposited through a SU-8 shadow mask to form the working electrode and counter electrode. A multi-walled carbon nanotube forest was grown directly on the silicon microneedle array and platinum nano-particles were electrodeposited. Silver was deposited on the Si microneedle array through another shadow mask and chlorinated to form a Ag/AgCl reference electrode. The 3-electrode electrochemical sensor was tested for various glucose concentrations in the range of 3∼20 mM in 0.01 M phosphate buffered saline (PBS) solution. The sensor's amperometric response to the glucose concentration is linear and its sensitivity was found to be 17.73 ± 3 μA/mM-cm^2^. This microneedle-based sensor has a potential to be used for painless diabetes testing applications.

## 1. Introduction

Diabetes is one of the most serious health problems mankind faces these days. It was estimated that people who have diabetes were 2.8% of the World population in 2000 but it is expected to rapidly increase over time [1]. The most common method of blood glucose monitoring is through sampling of a small amount of blood taken from the patient's fingertip using a tiny lancet. Frequent blood sampling from fingertips creates trypanophobia for many diabetes patients. There have been numerous investigations to develop alternative methods of glucose monitoring to avoid frequent fingertip blood sampling, including sampling from alternate sites other than the fingertip [2], monitoring tear fluid instead of blood droplets [3], non-invasive optical methods [4], and continuous glucose monitoring [5], among others.

Microelectromechanical systems (MEMS) technologies are ideally positioned to provide a variety of options for non-invasive or minimally invasive glucose monitoring. Iguchi *et al.* reported a wearable amperometric glucose sensor which was fabricated using soft MEMS techniques [6]. Paranjape *et al.* fabricated a polydimethylsiloxane (PDMS) dermal patch and used it for non-invasive transdermal glucose sensing [7]. Microneedles have been used for minimally invasive transdermal drug delivery [8]. It is also possible to use microneedles as minimally invasive body fluid sampling devices. Windmiller reported a biosensor based on a 3 × 3 pyramidal microneedle array which was loaded with a metallized carbon paste transducer [9]. Zimmermann *et al.* reported a hollow microneedle-based disposable minimally invasive self-calibrating enzymatic continuous glucose monitor [10].

The majority of glucose sensors are enzyme-based. Although glucose oxidase (GOx) is quite stable compared with other enzymes, it is known that GOx may lose its activity quickly when it is exposed to heat or unwanted chemicals. The performance of GOx-based glucose sensors is limited by electron transfer between enzyme and electrode because the catalytically active centers of GO_x_ are covered by a protein shell, therefore direct electron transfer from the enzyme to the electrode is difficult [11]. Common drawbacks of enzymatic glucose sensors are insufficient sensor data stability, complexity of the enzyme immobilization and oxygen limitation [12]. Recently, non-enzymatic glucose sensors have been studied as an alternative method to overcome the drawbacks of enzymatic glucose sensors. It is well known that nano-scale electrodes substantially increase the electroactive surface area, resulting in enhancement of electrocatalysis [13]. Park *et al.* reported a nanoporous platinum electrode-based glucose sensor which utilizes direct glucose oxidation on a greatly increased platinum surface area for improved amperometric current response [14].

The objective of this paper is to report a method of fabrication which seamlessly combines a microneedle array for potential transdermal body fluid extraction and a Pt nanoparticle embedded CNT array for greatly increased electroactive surface area for enhanced non-enzymatic electrochemical sensing. It also reports *in-vitro* glucose sensing characterization of the hierarchical micro/nano structured sensor.

## 2. Fabrication

**Figure 1.**
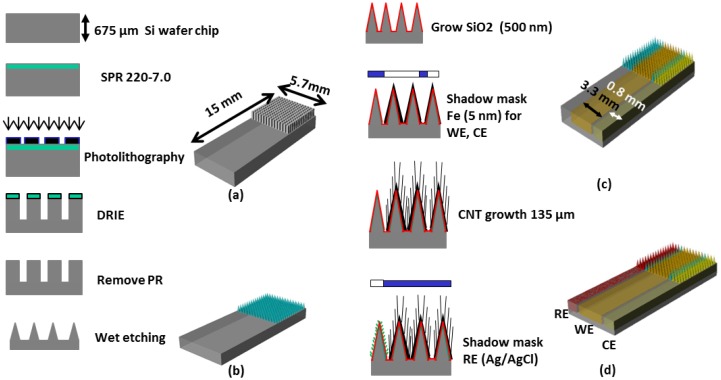
Fabrication sequence of the 3-electrode non-enzymatic microneedle-based glucose sensor (**a**) deep reactive ion etching of silicon to form rectangular pillar array; (**b**) wet etching of the rectangular pillar Si array to make sharp Si needle array; (**c**) iron deposition through a shaodw mask and MWCNT growth followed by Pt nano-particles electroplating; and (**d**) Ag deposition through a shadow mask and formation of Ag/AgCl reference electrode.

Figure 1 shows the overall fabrication sequence of the microneedle-based 3-electrode non-enzymatic sensor. A piece of Si wafer was cleaned and hexamethyldisilizane (HMDS) was spin coated. SPR 220-7.0 positive photoresist (MicroChem Corp., Newton, MA, USA) was then uniformly spin-coated and soft-baked. Ultraviolet (UV) exposure was carried out with an exposure dose of 470 mJ/cm^2^ then 2 h holding time was used for the photoresist to absorb ambient water vapor and permit the photoactive compound to react. The sample was then developed for 5 min. using MF-24A developer (MicroChem Corp.). A hard bake process was carried out in a convection oven at 180 °C for 5 min. to harden the photoresist and to utilize the photoresist as a mask for subsequent deep reactive ion etch (DRIE) process. DRIE of silicon was carried out by inductively coupled plasma (ICP) etcher to form 15 × 15 silicon pillar array (Figure 1a). The etch rate was approximately 1.5 μm/min. The resulting rectangular Si micro pillar was approximately 100 μm × 100 μm wide and 380 μm tall.

**Figure 2.**
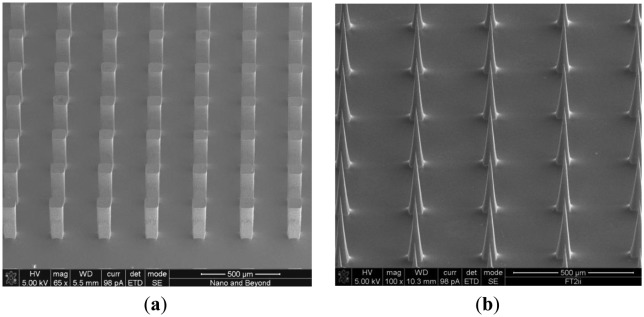
SEM images of (**a**) the 15 × 15 Si pillar array after DRIE; and (**b**) Si microneedle array after wet etch process. The scale bar shows 500 μm.

The Si pillar array was then isotropically wet etched in two steps using a mixture of hydrofluoric acid (HF) and nitric acid (HNO_3_) based on the work reported by Campbell *et al.* [15]. In the first wet etch step, a HF/HNO_3_ mixture container was placed on top of a magnetic hot plate and the sample was immersed in the HF/HNO_3_ wet etch solution. The sample was faced down and the etch solution was constantly agitated. In the second wet etch step, the sample was located at the bottom of the etch solution and was faced upward without agitation. This allows selective etching of the convex corner of the silicon pillar resulting in sharpened silicon tip (Figure 1b). Figure 2 shows scanning electron micrograph (SEM) images of the 15 × 15 Si pillar array after DRIE process (Figure 2a) and sharp Si microneedle array formation (Figure 2b). We analyzed the SEM images and found that Si needle is very sharp and the tip dimension is smaller than 1 μm.

**Figure 3.**
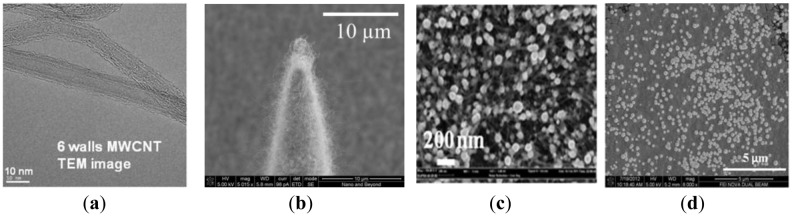
(**a**) A TEM image of the MWCNT grown on the microneedle array; (**b**) a SEM image of MWCNT forest grown near the tip of the non-planar Si microneedle array; (**c**) a SEM image showing well dispersed 50∼100 nm Pt nano-particles in the MWCNT forest by electrode position; and (**d**) a SEM image of the well dispersed 260 ± 20 nm AgCl nano-particles on the RE.

In our previous work on energy storage devices, we reported that CNTs with electrodeposited metal nanoparticles make an efficient electrode with high degree of electrical conductivity, mechanical stability, and reliability [16]. In order to greatly increase the electroactive surface area for non-enzymatic sensing, we directly grew a MWCNT array on the sharp silicon microneedle array and electrodeposited metallic nanoparticles. After formation of the sharp silicon microneedle array, a 500 nm thick SiO_2_ layer was conformally deposited by plasma enhanced chemical vapor deposition (PECVD). Then, 5 nm of iron catalyst was deposited using an electron beam evaporator in a selected area of the silicon microneedle array and the surrounding area through a shadow mask to create the working electrode (WE) and the counter electrode (CE). A vertically aligned approximately 135 μm MWCNT forest was then grown directly on iron catalyst-coated non-planar silicon microneedle array (Figure 1c). The details of the vertically-aligned MWCNT forest growth process can be found in [17]. Figure 3a shows a transmission electron microscopy (TEM) image of six walls of individual CNTs bundled together forming a MWCNT. The number of walls was determined after thorough analysis of the TEM images. Figure 3b shows a SEM image of the MWCNT forest grown near the sharp tip of the Si microneedle. The vertically aligned MWCNT forest was then collapsed by immersing the sample in ethanol. This process allows us to maintain the increased electrode surface of the MWCNT while minimizing any potential loss of the MWCNT bundles from the sharp Si microneedle-based electrode surface in the case the sensor was used for direct skin penetration and transdermal body fluid sampling. Pt nanoparticles were electrodeposited on the electrode surface using hexachloroplatinic acid hydrate (H_2_PtCl_6_) electroplating bath (Table 1). Figure 3c shows a SEM image of the well dispersed Pt nanoparticles in the MWCNT forest surface with sizes in the range of 50∼100 nm in diameter.

**Figure 4.**
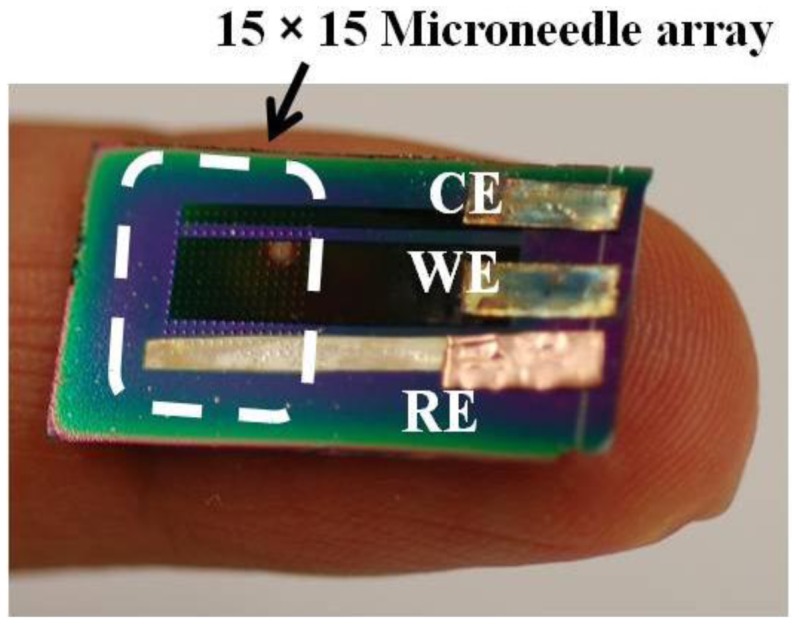
An optical image of the fabricated 3-electrode microneedle/CNT-combined electrochemical sensor.

To realize miniaturized electrochemical sensors with high accuracy, we integrated a reference electrode (RE) directly on the sensor. We chose Ag/AgCl as the RE material for this work due to the simplicity of fabrication. After the formation of WE and CE, a 100 nm thick titanium layer and a subsequent 300 nm thick silver layer were selectively deposited using another shadow mask to create the integrated RE. The silver layer was uniformly chlorinated (Figure 1d) in 1 M KCl/HCl buffer solution (pH 2.2, Table 2) with applied current of 1 μA for 1 min. Figure 3d shows a SEM image of the chlorinated silver electrode surface in which 260 ± 20 nm diameter AgCl nanoparticles grown on the Ag surface can be seen. Figure 4 shows an optical image of the fabricated hierarchical micro/nano microneedle/CNT-based electrochemical sensor. The 15 × 15 380 μm tall sharp Si microneedle array decorated with MWCNT and Pt nanoparticles is located in the indicated box. We designed the sensor in a manner that it has extended length of the electrodes beyond the 15 × 15 Si microneedle array region to potentially use it as a diabetes test strip in the future.

## 3. Characterization and Discussion

**Figure 5.**
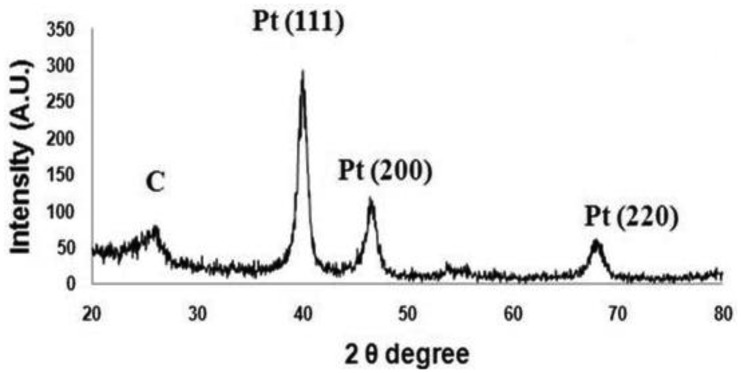
XRD pattern of the Pt nano-particles decorated MWCNT forest.

During the fabrication, X-ray diffraction (XRD) analysis was performed using an Ultima Ш (Rigaku Corp., Tokyo, Japan; instrument in the 2θ range from 20° to 80° to analyze Pt nano-particle deposition on the collapsed entangled MWCNT forest. Figure 5 shows an XRD pattern of the crystalline nature of Pt-CNT nanocomposites. Three major peaks at about 39.68°, 46.4° and 67.7° are believed to correspond to diffractions from the (111), (200), and (220) planes of the face-centered cubic lattice of platinum. Additionally, a feeble and a broad peak can be seen at around 26°, which can be attributed to the (002) planes of carbonized carbon. This result double confirms the presence of electrodeposited Pt nanoparticles in the collapsed entangled MWCNT forest.

**Figure 6.**
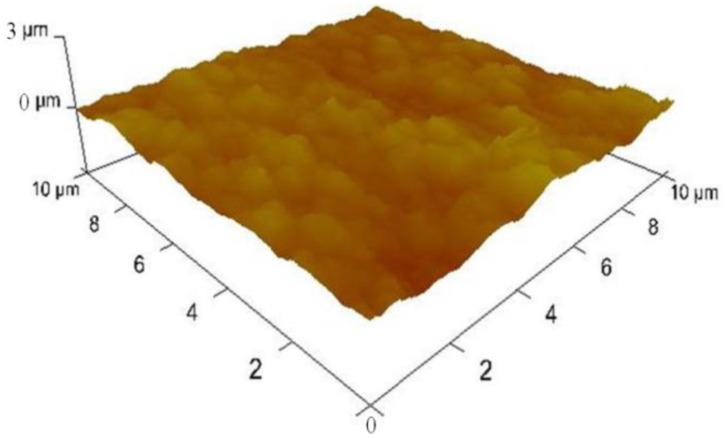
AFM data of the platinum nano-particles decorated MWCNT forest showing average surface roughness of 150 ± 16 nm.

We also inspected the surface morphologies of the samples during fabrication using a 3100 Dimension V Atomic Probe Microscope (Veeco, Plainview, NY, USA). Figure 6 shows an atomic force microscopy (AFM) image on the surfaces of Pt nanoparticles deposited MWCNT forest which revealed that the surface roughness was 149.7 ± 16 nm.

It shows a dramatic contrast from the surface roughness of plain platinum surface right after e-beam evaporation which was found to be 0.493 ± 0.3 nm. This result shows that the platinum nanoparticles electrodeposited on collapsed entangled MWCNT forest greatly increased the surface roughness. In addition to the outermost surface, we inspected SEM images and found that platinum nano-particles were well dispersed inside the entangled MWCNT forest.

**Figure 7.**
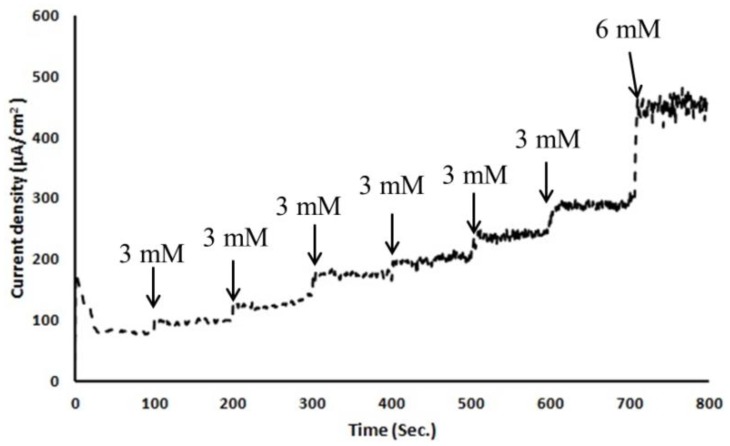
Chronoamperometry electrochemical response of the non-enzymatic microneedle glucose sensor as a function of glucose concentration in 0.01 M PBS solution at a +0.4 V potential *vs.* Ag/AgCl reference electrode.

To test this micro/nano hybrid structured sensor's performance, we carried out *in-vitro* glucose sensing characterization in 0.01 M pH 7.4 phosphate-buffered saline solution. Before any characterization, electrochemical solutions were thoroughly deaerated by purging with high-purity nitrogen gas for 30 min. For ease of handling of the 3-electrode-based electrochemical sensor, copper tape was attached as a connector between a potentiostat and the sensor. As shown in Figure 4, copper tape was directly overlapped on the patterned WE, CE and RE. However, the copper connection area was not immersed in PBS solution to keep the measured current responses only from the electrochemically reactive surface of the 3-electrode electrochemical sensor. Amperometric response of an electrode is generally studied by measuring the current response at a fixed potential by adding the known amount of analyte at regular intervals using chronoamperometry. All electrodes were thoroughly cleaned using deionized water before measurements and all potentials were referred to the integrated Ag/AgCl reference electrode. A detection potential of +0.4 V (*vs.* Ag/AgCl) was applied using a chronoamperometry setup to show the steady-state amperometric current responses of the microneedle-based non-enzymatic electrochemical sensor with various glucose concentrations in the range of 3∼20 mM in 0.01 M PBS solution over 800 s. As each additional 3 mM glucose was added to the test solution, current was nearly linearly increased (Figure 7). Noise in this chronoamperometry setup turned out to be minimal.

**Figure 8.**
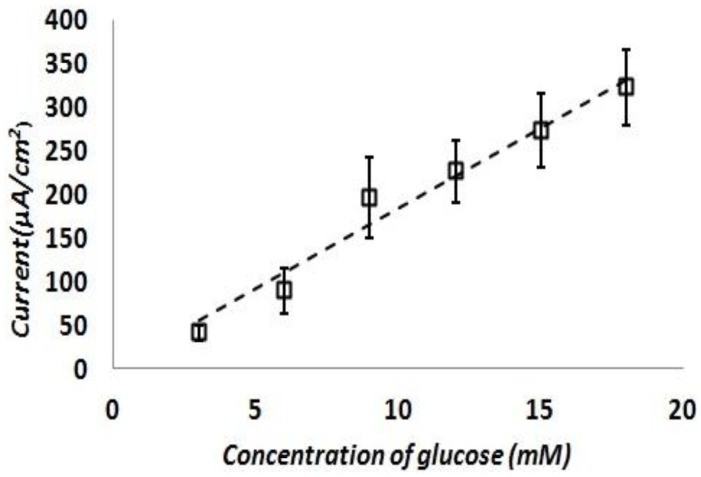
Current density responses of the 3-electrode glucose sensor as a function of the glucose concentration.

The test was repeated with fresh test solutions at least five times and current density response of the sensor was calculated. Figure 8 shows the calculated current density responses of the glucose sensor as a function of the glucose concentration. This result shows a reasonably linear response of the sensor at various glucose concentrations in the range of 3∼20 mM in 0.01 M PBS solution. The sensitivity of this sensor is turned out to be 17.73 ± 3 μA/mM-cm^2^. This sensitivity is on par or better than previously reported literature examples of similar non-enzymatic glucose sensing techniques as shown in Table 3.

## 4. Conclusions/Outlook

We have developed a fabrication process which uniquely combines a sharp silicon microneedle array and a MWCNT forest grown on a non-planar surface. We also have successfully demonstrated this microneedle-based Ag/AgCl reference electrode integrated 3-electrode electrochemical sensor as a non-enzymatic glucose sensor. This sensor has dramatically increased surface area due to the MWCNT forest and well dispersed platinum nanoparticles, resulting in high sensitivity. Since this sensor is microneedle-based, we envision that this sensor would potentially work as a minimally invasive finger-prick lancet high sensitivity glucose sensor as an alternative to common finger-prick lancet-based blood glucose sensors. We believe that the results reported in this paper pave the way toward developing a minimally invasive glucose sensor which should greatly improve the quality of life for those who need frequent blood sampling for their diabetes treatment.

## Figures and Tables

**Table 1.** Chemical composition of platinum electrodeposition bath.

**DI Water**	**300 mL**
H_2_PtCl_6_·xH_2_O	0.16 g (1.3 mM)
H_2_SO_4_	8 mL (0.5 mM)

**Table 2.** Chemical composition of 1 M Ag chlorinating solution (pH 2.2).

**DI Water**	**300 mL**
KCl	22.35 g
36.5% HCl	0.189 mL

**Table 3.** A comparison of glucose detection sensitivity of various non-enzymatic glucose sensors using different electrode types.

**Electrode**	**Sensitivity (μA/Mm−cm^2^)**	**Reference**
Au nanotube array	1.13	[18]
Pt nanoporous	1.65	[19]
Pt/MWCNTs	11.83	[20]
Pt/MWCNTs/Microneedle	17.73 ± 3	This work
